# The ion channel Trpc6a regulates the cardiomyocyte regenerative response to mechanical stretch

**DOI:** 10.3389/fcvm.2023.1186086

**Published:** 2024-01-08

**Authors:** Laura Rolland, Jourdano Mancilla Abaroa, Adèle Faucherre, Aurélien Drouard, Chris Jopling

**Affiliations:** Institute of Functional Genomics, University of Montpellier, CNRS, INSERM, LabEx ICST, Montpellier, France

**Keywords:** TRPC6 channel, heart regeneration, AP1 complex, mechanosensation, calcineurin/NFAT pathway

## Abstract

Myocardial damage caused, for example, by cardiac ischemia leads to ventricular volume overload resulting in increased stretch of the remaining myocardium. In adult mammals, these changes trigger an adaptive cardiomyocyte hypertrophic response which, if the damage is extensive, will ultimately lead to pathological hypertrophy and heart failure. Conversely, in response to extensive myocardial damage, cardiomyocytes in the adult zebrafish heart and neonatal mice proliferate and completely regenerate the damaged myocardium. We therefore hypothesized that in adult zebrafish, changes in mechanical loading due to myocardial damage may act as a trigger to induce cardiac regeneration. Based on this notion we sought to identify mechanosensors which could be involved in detecting changes in mechanical loading and triggering regeneration. Here we show using a combination of knockout animals, RNAseq and *in vitro* assays that the mechanosensitive ion channel Trpc6a is required by cardiomyocytes for successful cardiac regeneration in adult zebrafish. Furthermore, using a cyclic cell stretch assay, we have determined that Trpc6a induces the expression of components of the AP1 transcription complex in response to mechanical stretch. Our data highlights how changes in mechanical forces due to myocardial damage can be detected by mechanosensors which in turn can trigger cardiac regeneration.

## Introduction

Following a myocardial infarction, the loss of cardiac tissue results in significant changes in the mechanical loads exerted on the heart. As dynamic/elastic myocardium is replaced by relatively stiff non-contractile scar tissue, the resulting elevated preload and changes in tissue composition increases the amount of stretch exerted on the remaining myocardium, triggering an increased force of contraction and concomitant cardiomyocyte hypertrophy (Frank-Starling law) (1). While this adaptive mechanism initially compensates for the increased mechanical load, prolonged stress will become maladaptive and, without medical intervention, will ultimately result in pathological hypertrophy and heart failure.

In contrast to adult mammals, neonatal mice and adult zebrafish can fully regenerate their hearts after cardiac injury (2, 3). Rather than undergoing compensatory hypertrophy, neonatal mouse and adult zebrafish cardiomyocytes proliferate in response to the loss of myocardium. This suggests that the changes in mechanical loading which occur after cardiac injury in neonates/zebrafish may act as a trigger to induce cardiomyocyte proliferation and ultimately regeneration.

Although decades of research have been devoted to understanding the effects of mechanical stretch on non-proliferative adult cardiomyocytes there is very little information regarding the effects on cardiomyocytes which are capable of proliferation. However, *in vitro* evidence studying the effects of mechanical loading on embryonic mouse cardiomyocytes indicates an increase in the proliferation index following 24 h of cyclic stretch (4). Likewise, transcriptomic analysis of neonatal rat ventricular cardiomyocytes subjected to cyclic stretch *in vitro* showed a significant upregulation of genes associated with cell proliferation (5).

To understand whether increased mechanical stretch can induce cardiomyocyte proliferation *in vivo,* in models which are capable of cardiac regeneration, it is important to assess whether changes in mechanical force occur following cardiac injury. Analysis of adult zebrafish indicates that following cardiac injury, the initial response is similar to that observed in adult humans resulting in a stiffer myocardium due to the extensive fibrosis at the site of injury (6). Furthermore, the resulting volume overload initially induces an elongation of cardiomyocyte sarcomere length which reverts following cardiac regeneration. This indicates that following cardiac injury in adult zebrafish, the heart is subjected to significant changes in mechanical loading. Whether these forces can induce a proliferative response is at present unclear (6). However, other situations that can also increase cardiomyocyte stretch do support this hypothesis. In humans vigorous exercise results in an elevated preload resulting in increased cardiomyocyte stretch. These conditions trigger an adaptive hypertrophic response resulting in an essentially larger more powerful heart (1). On the other hand, in adult zebrafish, exercise can induce cardiomyocytes to proliferate rather than undergo hypertrophy (7). This indicates that increased mechanical load can act as a stimulus to induce cardiomyocyte proliferation. But care must be taken when extrapolating exercise induced cardiomyocyte hypertrophy vs. damage induced hypertrophy as it appears that different mechanisms are at play depending on the conditions (8). Understanding how increased stretch can influence cardiomyocyte proliferation, particularly *in vivo*, may provide invaluable information on how this phenomenon could be harnessed to induce a regenerative response.

Increased myocardial stretch can be sensed by a wide variety of mechanosensory mechanisms present in cardiomyocytes such as cell surface receptors, sarcomeric components, intercalated discs and stretch activated ion channels (1). The transient receptor potential (Trp) channels are a family of non-selective cation channels which can be regulated by a variety of stimuli including mechanical stretch. Of these, *TRPC3* and *TRPC6* are highly expressed in the heart and have been directly linked to pathological cardiac hypertrophy in response to chronic overload. In particular, stretch elevates intracellular Ca^2+^ which activates the CALCINEURIN/NUCLEAR FACTOR OF ACTIVATED T CELLS (NFAT) pathway triggering pathological hypertrophy and remodelling (9, 10). Both *TRPC3* and *TRPC6* have been shown to be responsible for the stretch induced increase in Ca^2+^ (9). Indeed, targeting both of these ion channels can inhibit pathological cardiomyocyte hypertrophy (11).

Because of the role TRPC6 plays in regulating the cardiomyocyte hypertrophic response to increased stretch in mammals we surmised that Trpc6 may also regulate cardiomyocyte proliferation in adult zebrafish regenerating hearts in response to the chronic mechanical overload associated with cardiac injury.

In this study we examined how the loss of *trpc6a* could affect cardiac regeneration in zebrafish. Similar to reports in mammals, we found that *trpc6a* knockout (KO) did not result in any observable cardiac developmental defects. However, adult *trpc6a* KO zebrafish failed to regenerate their hearts after 30 days following apical resection due to a reduction in cardiomyocyte proliferation. Transcriptomic analysis indicated that following cardiac injury, *trpc6a* KO zebrafish did not upregulate the expression of genes required for this process. In particular, this included orthologous components of the ACTIVATOR PROTEIN 1 (AP1) transcription factor complex which is critical for cardiac regeneration in adult zebrafish (12). Furthermore, we demonstrate that the stretch induced expression of AP1 components is markedly reduced in *trpc6a* KO zebrafish. Taken together these findings indicate that Trpc6a positively regulates the expression of pro-regenerative genes in response to mechanical stretch and that this process is required for successful cardiac regeneration.

## Results

### Loss of Trpc6a does not affect cardiac development

**Figure 1:**
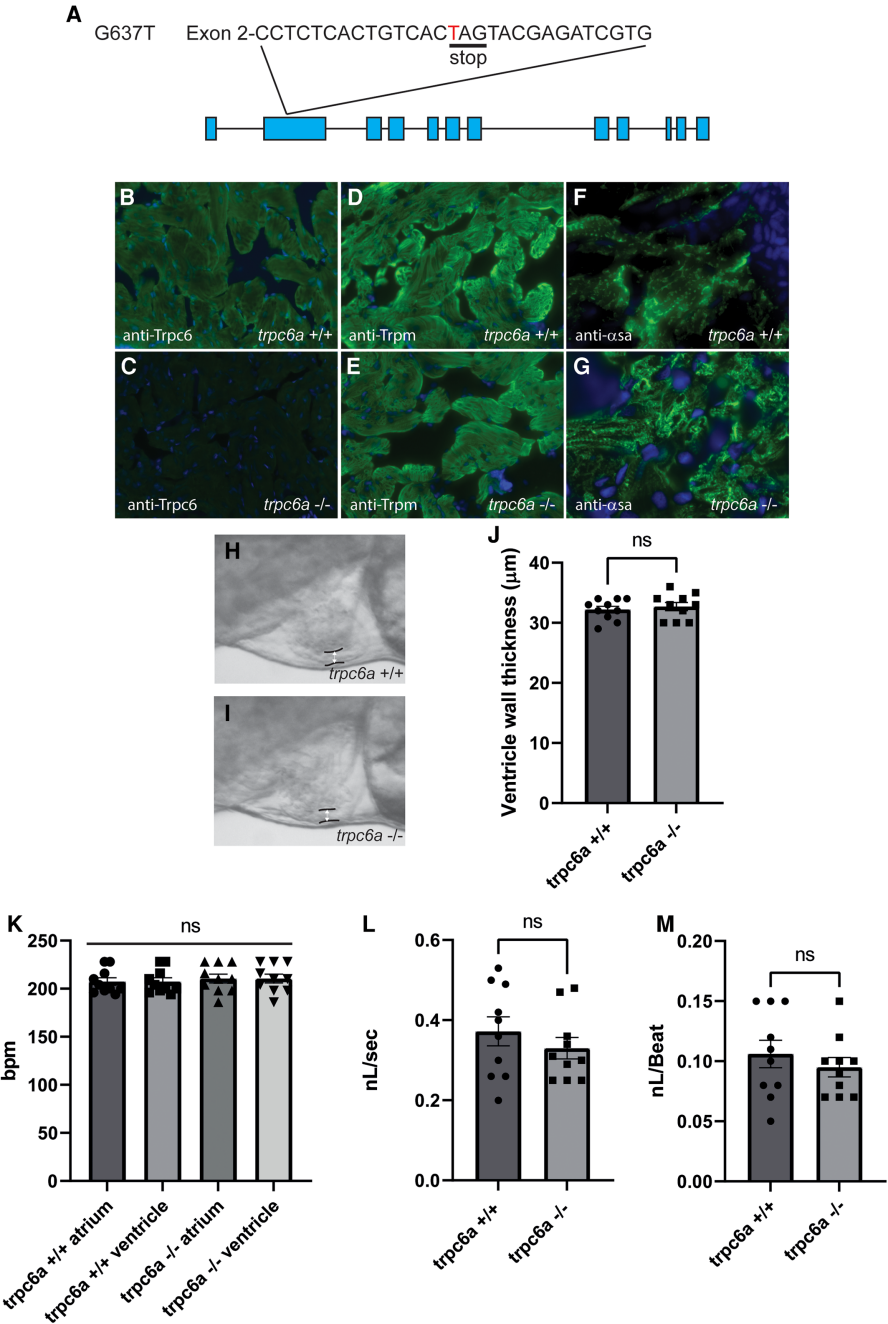
Loss of Trpc6a does not affect cardiac development. (**A**) Diagram of the point mutation carried by the sa23930 zebrafish transgenic line. The G637T mutation causes a premature stop codon in the Exon 2 of the *trpc6a*. (**B,C)** IHC images from adult heart sections showing the presence of Trpc6 in the myocardium of *trpc6a*^+/+^ but absent from the myocardium of *trpc6a*^−/−^ zebrafish. Trpc6: green, DAPI: blue. (**D,E)** IHC images from adult heart sections showing the organization of tropomyosin (Trpm) in myocardium of *trpc6a^+/+^* and in *trpc6a^−/−^* zebrafish. Trpm: green, DAPI: blue. (**F,G)**. IHC images from adult heart sections showing the organization of α-sarcomeric actin (α-sa) in the myocardium of *trpc6a^+/+^* and in *trpc6a^−/−^* zebrafish. α-sa: green, DAPI: blue. (**H,I**) Representative morphology of the ventricular wall of 5dpf larvae from control (*trpc6a*^+/+^, **H**) and trpc6a KO (*trpc6a^−/−^,* **I**) groups. (**J**) Ventricular wall thickness measurements of 5dpf larvae during diastole. *T*-test was used for statistical analysis. (**K**) Atrial and ventricular contraction rates (in bpm) of *trpc6a*^+/+^ and *trpc6a*^−/−^ 5dpf larvae. 1-way ANOVA was used for statistical analysis. (**L**) Blood flow velocity (in nL/s) measured in the caudal vein at 5dpf. *T*-test was used for statistical analysis. (**M**) Cardiac output (in nL/beat) of *trpc6a*^+/+^ and *trpc6a*^−/−^ 3dpf larvae. *t*-test was used for statistical analysis. (**H–M**) Data obtained on *n* = 10 larvae per group.

Zebrafish possess 2 paralogs of *TRPC6, trpc6a* and *trpc6b.* Previous research indicates that *trpc6a* is expressed in the developing heart of 5 days post fertilisation (dpf) larvae whereas *trpc6b* expression is restricted to motor neurons in the brain (13). Based on this data we focused on *trpc6a* as this paralog was likely to play a role similar to mammalian TRPC6 in the heart. To understand the role Trpc6a plays during cardiac development and regeneration, we utilised a KO zebrafish line which harbours a single base pair substitution G637T resulting in a premature stop codon in exon2 of *trpc6a* (Figure 1A). To confirm that this mutation results in a loss of Trpc6a protein, we performed immunohistochemistry (IHC) using a Trpc6 antibody. In this manner we could detect Trpc6 in the myocardium of adult *trpc6a^+/+^* zebrafish hearts (Figure 1B). In comparison, Trpc6 was absent in adult *trpc6a^−/−^* zebrafish hearts indicating that no functional Trpc6a protein was present in the KO line (Figure 1C). In parallel, we also assessed sarcomere structure in *trpc6a^−/−^* hearts using Tropomyosin (trpm) and α Sarcomeric actin (αsa) antibodies. We could not detect any observable differences in tropomyosin and sarcomeric actin labelling between *trpc6a^−/−^* and their *trpc6a^+/+^* siblings (Figures 1D–G). We next determined whether loss of Trpc6a affects cardiac development in zebrafish larvae as this could potentially impact processes occurring in adulthood, such as cardiac regeneration. Because of the role TRPC6 plays in hypertrophy in mammals we first measured the ventricular wall of 5dpf larvae (*n* = 10/group) (Figures 1H,I). In this manner we could not detect any significant differences between *trpc6a^−/−^* and *trpc6a^+/+^* larvae (Figure 1J). Furthermore, chamber morphology and looping appeared unaffected by the loss of *trpc6a* (data not shown). Mutations in *TRPC6* have also been associated with cardiac arrhythmias which could, if present, disrupt heart regeneration at later stages. Therefore, we analysed a variety of cardiac physiological parameters in both *trpc6a^−/−^* and *trpc6a^+/+^* larvae. Measurements of ventricular and atrial heart rates indicated there was no significant difference between *trpc6a^−/−^* and *trpc6a^+/+^* larvae (*n* = 10/group) (Figure 1K). Furthermore, we could not detect any blood regurgitation between the ventricle and atrium indicating that valve development occurred normally in *trpc6a^−/−^* larvae (data not shown). Lastly, we measured the blood flow rate and calculated the cardiac output in both *trpc6a^−/−^* and *trpc6a^+/+^* larvae. Our data indicates that there are no significant differences in these parameters between *trpc6a^−/−^* and *trpc6a^+/+^* larvae indicating that loss of Trpc6a does not appear to affect overall cardiac performance (*n* = 10/group) (Figures 1L,M). These data indicate that heart development and cardiac performance are not significantly affected in *trpc6a^−/−^* zebrafish larvae.

### Trpc6a is required for cardiac regeneration

**Figure 2:**
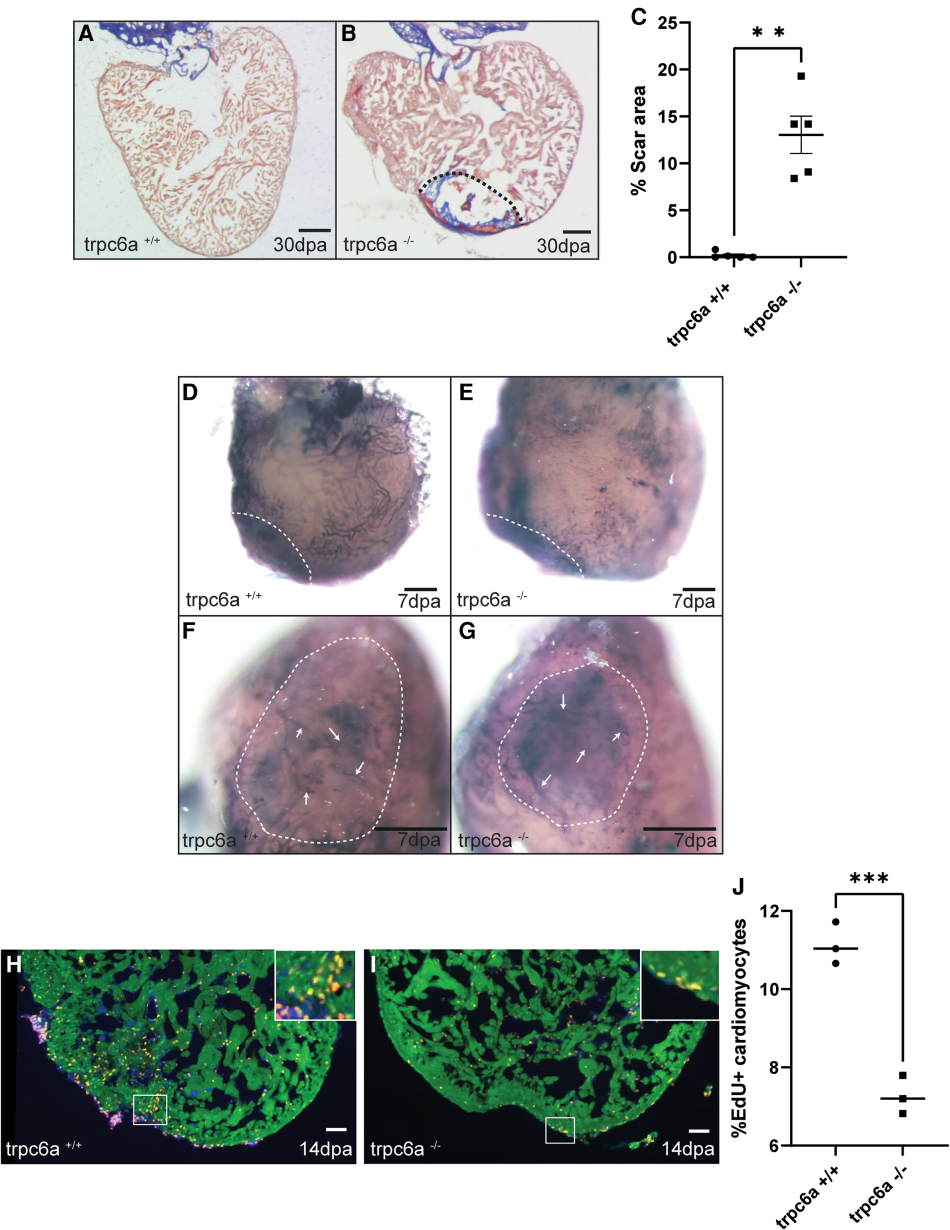
Trpc6a is required for cardiac regeneration. **(A-C**) AFOG staining images and quantification of the scar area at 30dpa. Representative image of AFOG staining obtained for *trpc6a^+/+^* (**A**) and *trpc6a^−/−^* (**B**) Scale bars: 200 μm. The dashed line outlines the scar region. (**C**) Histogram depicting the quantification of the scar area (*n*=5/group). Students *t*-test was used for statistical analysis. **: *p* value < 0.01. (**D–G**) Representative images of alkaline phosphatase staining showing the vasculature of 7dpa whole mount hearts. Low (**D,E**) and high (**F,G**) magnification of the vascular plexus present in the wound region of *trpc6a^+/+^* (**D,F**) and *trpc6a^−/−^* (**E,G**) zebrafish hearts. Scale bars: 200 μm. (**H–J**) Cardiomyocyte proliferation measured at 14dpa. Representative IHC images showing Mef2c (green), EdU (red) and DAPI (blue) for *trpc6a^+/+^* (**H**) and *trpc6a^−/−^* (**I**). The white box depicts a higher magnification image in the upper right corner. Scale bars: 100 μm. (**J)**. Quantification of EdU+ cardiomyocytes (*n*=3/group). Students *t*-test was used for statistical analysis. ***: *p* value < 0.001.

Because of the role TRPC6 plays in sensing changes in mechanical load after cardiac injury in mammals, we assessed whether the loss of Trpc6 affected cardiac regeneration in adult zebrafish. To achieve this, we performed apical resection of adult *trpc6a^−/−^* and *trpc6a^+/+^* zebrafish. At 30 days post amputation (dpa), histological staining using acid fuchsin orange G (AFOG) indicated that the loss of Trpc6a inhibited cardiac regeneration resulting in the presence of a significant fibrin/collagen scar (*n* = 5/group) (Figures 2A–C). Previous research indicates that TRPC6 can also play a role in angiogenesis (14). During cardiac regeneration in adult zebrafish, revascularization of the wound region is a critical early event which could be affected by the loss of Trpc6a. To address this possibility, we analysed wound revascularization at 7dpa. In this manner we could not detect any obvious differences in vascular plexus formation within the wound region of both *trpc6a^−/−^* and *trpc6a^+/+^* zebrafish hearts indicating that this process appears largely unaffected and is unlikely the cause of the defective regeneration we observed in *trpc6a^−/−^* hearts (*n* = 5/group) (Figures 2D–G). We next sought to determine whether cardiomyocyte proliferation had been affected in *trpc6a^−/−^* zebrafish. To meet this end, we preformed EdU labelling of resected *trpc6a^−/−^* and *trpc6a^+/+^* zebrafish hearts at 14dpa. Our analysis indicates that there is a significant reduction in the number of EdU labelled cardiomyocytes in the *trpc6a^−/−^* hearts compared to their *trpc6a^+/+^* siblings (*n* = 3/group) (Figures 2H–J). Taken together this data indicates that the loss of Trpc6a disrupts cardiac regeneration due to a significant reduction in cardiomyocyte proliferation.

### Transcriptomic analysis of *trpc6a* knockout hearts

**Figure 3:**
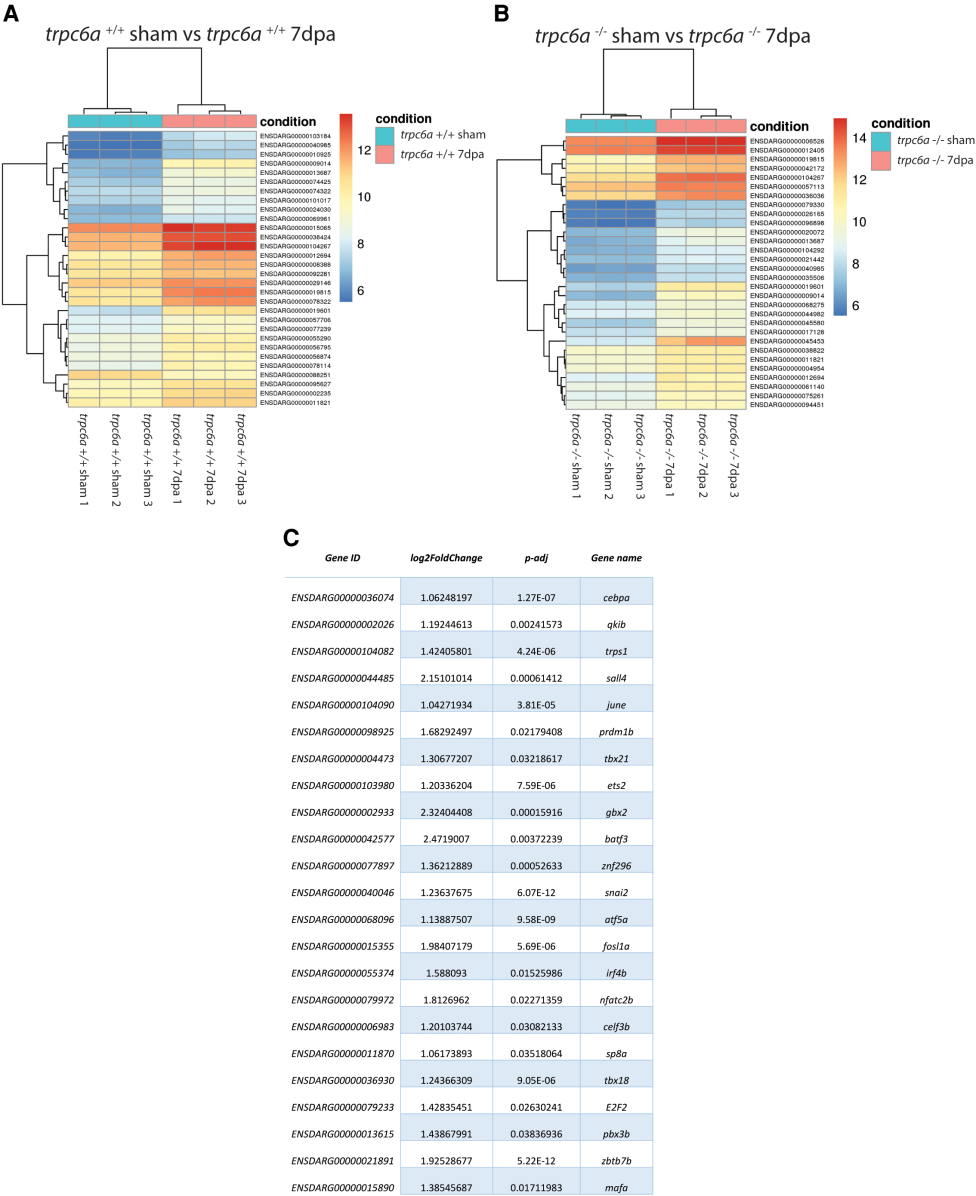
Loss of Trpc6a results in misregulated gene expression during regeneration. (**A,B**) Heatmaps showing the 30 top differentially regulated genes between sham and 7dpa hearts of *trpc6a^+/+^* (**A**) and *trpc6a^−/−^* (**B**) zebrafish (**C)** Table of transcription factors which are significantly upregulated in *trpc6a^+/+^* hearts but not in *trpc6a^−/−^* hearts following injury (compared to their respective sham controls).

To determine what effect the loss of Trpc6a had on the cardiac transcriptome during heart regeneration, we performed bulk RNA sequencing of sham operated and 7dpa resected *trpc6a^−/−^* and *trpc6a^+/+^* hearts (*n* = 5/group). Bi-clustering heatmaps were generated to visualize the expression profile of the top 30 differentially expressed genes sorted by their adjusted *p*-value by plotting their log2 transformed expression values (Figures 3A,B). Analysis of sham vs. 7dpa samples indicates that there are 515 differentially expressed genes (DEGS) specific to 7dpa *trpc6a^+/+^* regenerating hearts compared with 451 DEGS specific to 7dpa *trpc6a^−/−^* hearts. We next performed GO analysis of the DEGS specific to either *trpc6a^+/+^* 7dpa hearts or *trpc6a^−/−^* 7dpa hearts (Tables 1, 2). From this data we were able to determine that there are numerous pathways which are associated with regenerating *trpc6a^+/+^* 7dpa hearts (30 in total) including a number of signalling pathways (Adipocytokine, PPAR, Toll-like receptor, NOD-like receptor, C-type lectin, Insulin, MTOR, TGF-beta and VEGF), metabolic processes and the cell cycle (Table 1). In contrast there are 15 pathways associated with *trpc6a^−/−^* 7dpa hearts, these include a different set of signalling pathways (Hedgehog, FoxO, Apelin and Adrenergic) and, notably, a lack of cell cycle pathways (Table 2). We next focused on the DEGS which were specific for regenerating *trpc6a^+/+^* hearts as these likely include genes which may be induced by Trpc6a activation. In this manner we identified a number of transcription factors which were significantly upregulated in the *trpc6a^+/+^* hearts but not in *trpc6a^−/−^* hearts (Figure 3C). Of particular interest were components of the AP1 transcription factor complex (*june* and *fosl1a*), a critical regulator of cardiac regeneration in adult zebrafish and a known downstream target of Trpc6 (Figure 3C) (15). We next questioned whether the upregulation of *june* and *fosl1a* was a common mechanism associated with cardiac injury which also occurred in adult mammals. To address this, we reanalysed single nuclei RNAseq (snRNAseq) data performed on infarcted adult mouse hearts from Yamada et al. (16). Although adult mice do not appear to possess a *june* orthologue we were able to determine that the expression of *Fosl1* did not change significantly in border zone cardiomyocytes following myocardial infarction in adult mice (Supplementary Figures S1B,C). This indicates that the upregulation of *fosl1a* we observed is likely specific to regenerating zebrafish hearts. Trpc6 also classically induces calcineurin/NFAT signalling (10) and in this respect we observed a significant increase in expression of *nfatc2b* specifically in regenerating *trpc6a^+/+^* hearts (Figure 3C and Supplementary Figure S2A) in conjunction with a significant increase in expression of the downstream target *nppb* (*p*-adj 1.87E-05) (Supplementary Figure S3A). In comparison, in adult mice *Nfatc2* does not appear to be significantly upregulated by cardiomyocytes following myocardial infarction (Supplementary Figures S2B,C), however there is a significant increase in the cardiomyocyte expression of *Nppb* in infarcted mouse hearts (Supplementary Figures S3B,C). Taken together, this indicates that the calcineurin/NFAT signalling pathway appears to be activated by Trpc6a in regenerating zebrafish hearts. Lastly, we also assessed whether *trpc6a* expression is upregulated during regeneration. Analysis of *trpc6a^+/+^* sham vs. 7dpa samples indicates there is a significant upregulation of *trpc6a* expression during regeneration which is not the case in the *trpc6a^−/−^* samples (Supplementary Figure S4A). In contrast adult mice do not appear to upregulate the expression of Trpc6 following myocardial infarction (Supplementary Figures S4D,E). We subsequently performed IHC for Trpc6a on regenerating *trpc6a^+/+^* hearts in an attempt to localise the upregulation in expression (Supplementary Figures S4B,C). Although there appears to be a moderate elevation of Trpc6a in the cardiomyocytes adjacent to the wound region compared to distal regions (Supplementary Figures S4B,C) we were unable to quantify this satisfactorily and this will require higher resolution analysis such as single nuclei RNA sequencing.

**Table 1:** GO analysis of DEGS specific to 7dpa *trpc6a^+/+^* hearts.

Enrichment FDR	nGenes	Pathway genes	Fold enrichment	Pathway	URL	Genes
5.05E-05	16	204	4.619026	Cytokine-cytokine receptor interaction	http://www.genome.jp/kegg-bin/show_pathway?dre04060	tnfb amh bmp6 ifng1 il13ra2 xcr1a.1 cd40lg ACVR1C tnfsf13 cxcl11.5 cxcl11.6 cxcl11.8 il1b cxcl11.1 il6r cd27
6.29E-05	58	1,762	1.938575	Metabolic pathways	http://www.genome.jp/kegg-bin/show_pathway?dre01100	acsl1b elovl5 lta4h abat odc1 hprt1 ptgs2b acsl4b cbsb cahz gpt2 galnt6 ca2 rimkla dgat2 fads2 gpt2l acp5a lpin1 tph1a tkta cmpk2 scd gldc gatm tpk1 qdpra enpp6 impdh1a alox5b.2 gpx3 gfpt2 entpd1 pygma pipox adcy2a rdh10a gyg1b mt-nd3 agpat4 ckmt2a acod1 bpgm nt5e atp6v1ab b4galnt1b hpse2 acsl2 b3galt1a st6gal2b smpd3 pkmb elovl1a cyp24a1 dgat1a kmt5ab cox7c gcshb
0.003674	6	46	7.681641	Glycine, serine and threonine metabolism	http://www.genome.jp/kegg-bin/show_pathway?dre00260	cbsb gldc gatm pipox bpgm gcshb
0.003674	8	89	5.293715	Adipocytokine signaling pathway	http://www.genome.jp/kegg-bin/show_pathway?dre04920	acsl1b acsl4b socs3b irs1 pparab irs2b acsl2 mapk10
0.00659	7	76	5.424316	Fatty acid metabolism	http://www.genome.jp/kegg-bin/show_pathway?dre01212	acsl1b elovl5 acsl4b fads2 scd acsl2 elovl1a
0.009666	10	165	3.569247	NOD-like receptor signaling pathway	http://www.genome.jp/kegg-bin/show_pathway?dre04621	mapk12b cyba tlr4ba stat2 gbp1 ctsba ripk3 sting1 il1b mapk10
0.01402	7	91	4.530198	PPAR signaling pathway	http://www.genome.jp/kegg-bin/show_pathway?dre03320	acsl1b acsl4b fads2 scd fabp7b pparab acsl2
0.021301	7	100	4.12248	Toll-like receptor signaling pathway	http://www.genome.jp/kegg-bin/show_pathway?dre04620	mapk12b tlr4ba cxcl11.5 cxcl11.6 cxcl11.8 il1b mapk10
0.022013	10	194	3.0357	Apoptosis	http://www.genome.jp/kegg-bin/show_pathway?dre04210	tuba2 ctsba gzm3.2 prf1.9 tuba4l baxa gzm3.3 mapk10 ptpn13 gadd45ab
0.024993	4	34	6.928539	Biosynthesis of unsaturated fatty acids	http://www.genome.jp/kegg-bin/show_pathway?dre01040	elovl5 fads2 scd elovl1a
0.024993	5	56	5.258266	Ferroptosis	http://www.genome.jp/kegg-bin/show_pathway?dre04216	acsl1b acsl4b slc3a2a slc7a11 acsl2
0.025784	9	173	3.063776	Insulin signaling pathway	http://www.genome.jp/kegg-bin/show_pathway?dre04910	prkar2ab socs3b socs1a rapgef1a irs1 pygma irs2b socs1b mapk10
0.038754	8	153	3.07935	Cell adhesion molecules	http://www.genome.jp/kegg-bin/show_pathway?dre04514	ptprfb ntng1a cldn11a selp itgb7 cldn2 cldni cd40lg
0.039034	3	21	8.413225	Fatty acid biosynthesis	http://www.genome.jp/kegg-bin/show_pathway?dre00061	acsl1b acsl4b acsl2
0.041225	7	126	3.27181	C-type lectin receptor signaling pathway	http://www.genome.jp/kegg-bin/show_pathway?dre04625	mapk12b ptgs2b stat2 bcl10 nfatc2b il1b mapk10
0.054554	4	47	5.012134	Alanine, aspartate and glutamate metabolism	http://www.genome.jp/kegg-bin/show_pathway?dre00250	abat rimkla gpt2l gfpt2
0.055277	4	48	4.907715	ABC transporters	http://www.genome.jp/kegg-bin/show_pathway?dre02010	abcc12 abcb6b abca1a abca1b
0.093427	8	189	2.492808	Necroptosis	http://www.genome.jp/kegg-bin/show_pathway?dre04217	tlr4ba ifng1 stat2 pygma baxa ripk3 il1b mapk10
0.105821	4	60	3.926172	Arachidonic acid metabolism	http://www.genome.jp/kegg-bin/show_pathway?dre00590	lta4h ptgs2b alox5b.2 gpx3
0.124548	6	131	2.69737	TGF-beta signaling pathway	http://www.genome.jp/kegg-bin/show_pathway?dre04350	amh bmp6 ifng1 hjv id4 ACVR1C
0.124548	3	37	4.775074	Intestinal immune network for IgA production	http://www.genome.jp/kegg-bin/show_pathway?dre04672	itgb7 cd40lg tnfsf13
0.150773	4	70	3.36529	Glycerolipid metabolism	http://www.genome.jp/kegg-bin/show_pathway?dre00561	dgat2 lpin1 agpat4 dgat1a
0.170398	3	44	4.015403	Cytosolic DNA-sensing pathway	http://www.genome.jp/kegg-bin/show_pathway?dre04623	ripk3 sting1 il1b
0.170398	7	184	2.240479	Herpes simplex virus 1 infection	http://www.genome.jp/kegg-bin/show_pathway?dre05168	tnfb ifng1 socs3b stat2 baxa sting1 il1b
0.172247	4	78	3.020132	VEGF signaling pathway	http://www.genome.jp/kegg-bin/show_pathway?dre04370	prkcg mapk12b ptgs2b nfatc2b
0.172247	8	229	2.057383	Tight junction	http://www.genome.jp/kegg-bin/show_pathway?dre04530	cldn11a cldn2 tuba2 cldni cgna tjp2a tuba4l mapk10
0.172247	10	314	1.87556	Salmonella infection	http://www.genome.jp/kegg-bin/show_pathway?dre05132	mapk12b tlr4ba cyth4a tuba2 tuba4l baxa ripk3 flnb il1b mapk10
0.173084	6	152	2.324707	Cell cycle	http://www.genome.jp/kegg-bin/show_pathway?dre04110	cdkn1ca wee2 ttk si:ch211-160f23.5 ccne2 gadd45ab
0.178565	2	21	5.608817	Nitrogen metabolism	http://www.genome.jp/kegg-bin/show_pathway?dre00910	cahz ca2
0.1873	7	198	2.082061	MTOR signaling pathway	http://www.genome.jp/kegg-bin/show_pathway?dre04150	prkcg rragd lpin1 sgk1 slc3a2a irs1 atp6v1ab

**Table 2:** GO analysis of DEGS specific to 7dpa *trpc6a^−/−^* hearts.

Enrichment FDR	nGenes	Pathway genes	Fold enrichment	Pathway	URL	Genes
0.017232	46	1,762	1.757132	Metabolic pathways	http://www.genome.jp/kegg-bin/show_pathway?dre01100	inppl1b phgdh pde4cb kmo abo ptges fbp1b gucy2f csad mboat1 prxl2b ampd2b hsd20b2 pde4a gla dnmt3bb.1 dctd galca acaa2 arg2 atp6ap1a tyms dck dhrs3a ugt2a4 dnmt3ba shmt1 entpd5a cers6 aldh9a1a.2 alox5a plcd4a hyal2a dgkg irg1l gck nos1 cyp3c4 gpam cyp26b1 vkorc1 prodha st8sia1 gart fah mgst3a
0.03084	6	69	5.852679	Hedgehog signaling pathway	http://www.genome.jp/kegg-bin/show_pathway?dre04340	prkacba gli2b dhh hhip boc bcl2b
0.0529	5	70	4.807557	Glycerolipid metabolism	http://www.genome.jp/kegg-bin/show_pathway?dre00561	mboat1 gla aldh9a1a.2 dgkg gpam
0.0529	3	20	10.09587	Glycosphingolipid biosynthesis	http://www.genome.jp/kegg-bin/show_pathway?dre00603	abo gla st8sia1
0.0529	3	20	10.09587	One carbon pool by folate	http://www.genome.jp/kegg-bin/show_pathway?dre00670	tyms shmt1 gart
0.0529	4	38	7.084821	DNA replication	http://www.genome.jp/kegg-bin/show_pathway?dre03030	mcm3 mcm4 dna2 mcm2
0.0529	7	131	3.596493	TGF-beta signaling pathway	http://www.genome.jp/kegg-bin/show_pathway?dre04350	thbs1b dcn rgma inhbab id2b gdf7 ltbp1
0.0529	4	37	7.276303	Intestinal immune network for IgA production	http://www.genome.jp/kegg-bin/show_pathway?dre04672	ccr9a cxcr4a ccl25b ccl27b
0.115985	7	158	2.981903	Purine metabolism	http://www.genome.jp/kegg-bin/show_pathway?dre00230	pde4cb gucy2f ampd2b pde4a dck entpd5a gart
0.12684	8	204	2.639443	Cytokine-cytokine receptor interaction	http://www.genome.jp/kegg-bin/show_pathway?dre04060	inhbab gdf7 ccr9a cxcr4a ccl25b il7r ccl27b cxcl8b.3
0.152687	4	66	4.07914	Arginine and proline metabolism	http://www.genome.jp/kegg-bin/show_pathway?dre00330	arg2 aldh9a1a.2 nos1 prodha
0.152687	7	176	2.676935	FoxO signaling pathway	http://www.genome.jp/kegg-bin/show_pathway?dre04068	bnip4 ccnb3 klf2a foxg1c s1pr4 il7r agap2
0.164147	7	185	2.546706	Apelin signaling pathway	http://www.genome.jp/kegg-bin/show_pathway?dre04371	prkacba myl4 gnai2b agtr1a mef2ca klf2a nos1
0.164147	6	142	2.843907	Melanogenesis	http://www.genome.jp/kegg-bin/show_pathway?dre04916	prkacba creb3l1 gnai2b edn1 ednrbb wnt16
0.173124	8	233	2.310929	Adrenergic signaling in cardiomyocytes	http://www.genome.jp/kegg-bin/show_pathway?dre04261	prkacba myl4 creb3l1 gnai2b agtr1a tnnt2e bcl2b ppp2r2bb

### Trpc6a regulates the stretch induced expression of Ap1 transcription factor components

**Figure 4:**
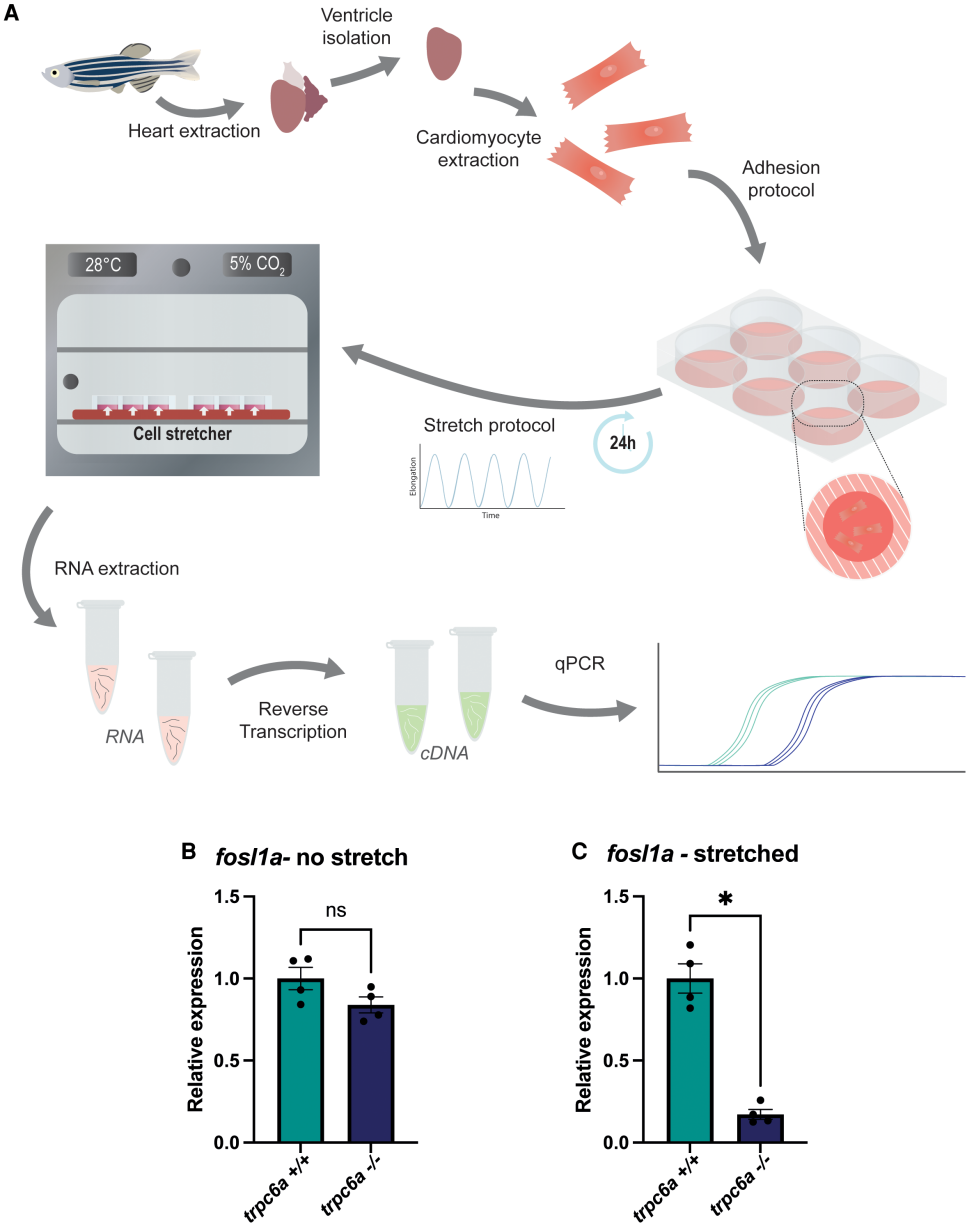
Trpc6a regulates the stretch induced expression of AP1 transcription factor components. (**A**) Schematic representation of the experimental design. Cardiomyocytes were isolated from extracted hearts and plated onto poly lysine-coated plates. A cyclic stretch protocol was applied for 24 h before RNA extraction and RT-qPCR. (**B**) Relative expression of *fosl1a* in unstretched *trpc6a^+/+^* and *trpc6a^−/−^* cardiomyocytes. (**C**) Relative expression of *fosl1a* in *trpc6a^+/+^* and *trpc6a^−/−^* cardiomyocytes subjected to cyclic stretch. Mann–Whitney test was used for statistical analysis. *: *p* value < 0.05.

Previous data indicates that pathologically stretching cardiomyocytes *in vivo* activates TRPC6, which in turn induces downstream gene expression (11). Based on this, we assessed whether Trpc6a regulated the expression of the AP1 transcription factor component *fosl1a* in response to mechanical stretch. To achieve this, we dissociated and isolated cardiomyocytes from either *trpc6^−/−^* or *trpc6a^+/+^* hearts and subjected them to 24 h of cyclic stretch (*n* = 5 hearts per group, 3 groups per condition) (Figure 4A). Following the completion of this protocol, we harvested the cardiomyocytes and performed RT qPCR for both *fosl1a* and *nppb* (Figure 4A). In this manner, we determined that under static conditions there is no significant difference in the relative expression of *fosl1a* between *trpc6a^+/+^* cardiomyocytes when compared to *trpc6^−/−^* cardiomyocytes. However, under stretch conditions the expression of *fosl1a* was significantly greater in *trpc6a^+/+^* cardiomyocytes when compared to *trpc6^−/−^* cardiomyocytes (Figure 4B,C). These data indicate that loss of Trpc6a in cardiomyocytes results in a failure to upregulate the expression of the AP1 transcription factor component *fosl1a.* We also assessed the expression of the downstream target of calcineurin/NFAT signalling, *nppb*. Although there appears to be a similar trend in the expression of *nppb* in response to mechanical stretch this was not significant (Supplementary Figures S5A,B).

## Discussion

The TRP ion channel TRPC6 is responsible for detecting increased mechanical stretch in cardiomyocytes and activating the CALCINEURIN/NFAT pathway (10). Under pathophysiological conditions of chronic elevated cardiomyocyte stretch, for example volume overload caused by a cardiac ischemia, this will lead to pathological hypertrophy and ultimately heart failure (10). Whether TRPC6 could also play a role in detecting increased cardiomyocyte stretch and triggering cardiac regeneration in animal models capable of this feat is currently unknown.

The results of this study indicate that Trpc6a is an essential component of the cardiac regenerative response in adult zebrafish. Our data demonstrates that loss of Trpc6a results in a failure to regenerate the heart after 30 days following cardiac resection. Early regenerative processes such as revascularization appear largely unaffected, however cardiomyocyte proliferation is significantly impeded in the absence of Trpc6a signaling leading to the persistence of extensive scarring. Furthermore, comparative transcriptomic analysis of *trpc6a^−/−^* and *trpc6a^+/+^* resected hearts indicates that loss of Trp6a substantially impacts gene expression. In particular components of the AP1 transcription factor complex, which are required for successful cardiac regeneration, are not upregulated in the absence Trpc6a. Lastly, our data indicates that Trpc6a regulates the expression of AP1 transcription factor complex components in response to mechanical stretch. Together these results indicate that, in adult zebrafish, increased/chronic cardiomyocyte stretch associated with cardiac injury is sensed by Trpc6a which subsequently activates downstream signaling pathways resulting in the expression of genes, such as AP1 transcription factor complex components, which are involved in driving cardiac regeneration.

### The Frank-Starling law and cardiac hypertrophy

The Frank-Starling law was described over a century ago and explains how elevated ventricular preloading, which stretches cardiomyocytes, results in an increased force of contraction in order to maintain circulatory homeostasis (17). Stretching cardiomyocytes increases the calcium sensitivity of their sarcomeres resulting in enhanced contractility. In situations where ventricular preloading is maintained, there is a further progressive increase in the force of contractility termed the slow force response (SFR) which is driven by elevated, TRPC3 and TRPC6 dependent (9), Ca^2+^ transients (18).

Chronic ventricular loading, for example after myocardial ischemia, results in cardiac remodeling and pathological cardiomyocyte hypertrophy leading, ultimately, to heart failure. The molecular mechanisms which drive pathological cardiac hypertrophy (as opposed to physiological hypertrophy induced by exercise) are largely driven by CALCINEURIN and its downstream effector NFAT (8). Chronic increases in mechanical load result in elevated intracellular Ca^2+^ which in turn activates CALCINEURIN. CALCINEURIN subsequently dephosphorylates NFAT which translocates into the nucleus and regulates the expression of genes which drive pathological hypertrophy.

### The role of Trpc6 in cardiac pathology

The ion channels TRCP3 and TRPC6 are responsible for detecting increases in cardiomyocyte stretch and generating the sustained Ca^2+^ transients which drive this pathological process (10). It is apparent then that TRPC6 plays a central role in the cardiac mechanosensitive response to volume overload which results in pathological hypertrophy. Conversely, our data indicates that, in adult zebrafish, it appears that the response to volume overload regulated by Trpc6a results in cardiomyocyte proliferation and ultimately cardiac regeneration. In mice, global KO of *Trpc6* results in increased mortality after myocardial infarction, however this is primarily due to the role Trpc6 plays in cardiac fibroblast activation, a process which is essential for early scar formation in order to avoid cardiac rupture (19). Blood pressure in adult zebrafish is around 50 times lower than mice (2.5 mmHg vs. 100 mmHg) (20) and as such the formation of a clot is sufficient to avoid excessive blood loss following damage to the myocardium. Because of the role TRPC6 plays in different cell types in mammals, it will be interesting to determine the effect that conditional, cardiomyocyte specific, deletion of *Trpc6* has following myocardial infarction*.* Furthermore, due to the close relationship between TRPC3 and TRPC6 it will also be interesting to assess whether *trpc3* also plays a role in cardiac regeneration in zebrafish.

### The TRPC6-CALCINEURIN-NFAT axis

By taking the opposite approach to genetic KO, constitutive, cardiomyocyte specific, over-expression of *Trpc6* in adult mice activates the CALCINEURIN/NFAT pathway resulting in pathological hypertrophy and lethality (10). However, it would also be of interest to assess what effect *Trpc6* overexpression has at earlier stages of development when cardiomyocytes are capable of proliferating and regenerating damaged myocardium. Downstream of TRPC6, cardiomyocyte specific overexpression of an active-CALCINEURIN isoform in adult mice is sufficient to trigger pathological hypertrophy and heart failure (21). Furthermore, similar experiments performed in neonatal mice indicates that active-CALCINEURIN induces a switch from proliferation to hypertrophic growth in cardiomyocytes which are normally hyperplastic at this stage of development (22). While these data seem at odds with our finding that Trpc6a plays a beneficial role during cardiac regeneration, our transcriptomic data indicates that other Trpc6a mediated mechanisms are also involved.

### Trpc6 and the AP1 transcription factor complex

Although much focus has been placed on the TRPC6-CALCINEURIN-NFAT axis, TRPC6 also activates other signalling mechanisms such as AP1 mediated gene transcription (15). We found that, during cardiac regeneration, there is a significant increase in the expression the AP1 transcription factor components *june* and *fosl1a* which does not occur when Trpc6a is absent. This is in-line with previous *in vitro* data indicating that activation of Trpc6 results in increased *c-fos* expression (23). More recently, the AP1 transcription factor complex has been shown to be a critical regulator of cardiac regeneration in adult zebrafish (12). In particular, cardiomyocyte specific expression of a dominant-negative *Fos* isoform significantly inhibits cardiac regeneration. This loss of AP1 function results in defective cardiomyocyte sarcomere disassembly and proliferation and also affects their ability to extend protrusions into the site of injury (12). Interestingly, although the AP1 components *JunB* and *Fosl1* are upregulated in adult zebrafish hearts after injury, the same is not true for adult mice after myocardial infarction (12, 24). Furthermore, overexpression of *JunB* and *Fosl1* in neonatal rat cardiomyocytes is sufficient to induce proliferation and protrusive behaviour in these cells similar to that observed in zebrafish cardiomyocytes (12). These data indicate a difference in AP1 signalling between adult zebrafish and mammals which may partly explain the differences we observed following KO of *trpc6a*. Lastly, we have demonstrated that Trpc6a regulates the expression of the AP1 transcription factor component *fosl1a* in response to mechanical stretch, similar to reports in mammalian cardiomyocytes (5, 25). Although care must be taken when extrapolating *in vitro* data, it is likely that this is also the situation which occurs during cardiac regeneration in adult zebrafish. The increase in myocardial stretch caused by volume overload following cardiac injury could activate Trpc6a and induce the expression of AP1 components required for cardiac regeneration. In summary we have identified Trpc6a as a critical regulator of cardiac regeneration. Furthermore, we also demonstrate that Trpc6a can induce the expression of AP1 components in response to mechanical stretch *in vitro*. Future studies will be required to establish exactly why Trpc6a induces a regenerative response in adult zebrafish compared to the pathological response in mammals and also whether this is restricted to cardiomyocytes or also involves other cell types which express TRPC6 such as cardiac fibroblasts.

## Methods

### Zebrafish line and husbandry

*Trpc6a* KO G637T (sa23930) zebrafish line was purchased from ZIRC and maintained under standardized conditions (26). The sa23930 Trpc6a KO was established and maintained on the ABWT strain. Experiments were conducted in accordance with local approval and the European Communities council directive 2010/63/EU. A mixture of males and females were used in all experiments using adult zebrafish.

### Larval heart rate and blood flow analysis

5dpf larvae were anaesthetised and mounted in low melt agarose. 30 s videos of either cardiac contractions or blood flow were recorded using a Point Grey GRAS-03K2C-C high speed camera. Heart rate and blood flow were analysed using ViewPoint MicroZebraLab software and ImageJ software. *T*-test and ANOVA statistical analysis was performed using GraphPad Prism.

### Resection

Cardiac resection were performed on 6–10-month-old zebrafish as previously described (2), in accordance with local approval (APAFIS#2021021117336492 v5).

### Immunohistochemistry and histological staining

Immunohistochemistry and histological staining were performed on 10 *μ*m heart sections as previously described (27). The antibodies used in this manuscript are listed below:

anti-Trpc6 (OST00081W, Osenses)

anti-Trpm (T2780, Sigma)

anti-Mef2c (ab197070 Abcam)

anti-α-Sarcomeric actin (A2172, Sigma)

EdU labelling was performed according to the manufacturer's instructions (Click-iT EdU Kit C10337, Molecular Probes). Acid Fuchsin-Orange G (AFOG) staining was performed as previously described (28) and the size of the scar area was calculated using ImageJ software. *T*-test statistical analyses was performed using GraphPad Prism. Alkaline phosphate staining was performed on whole-mount heart as previously described (27).

### Imaging

A Zeiss Discovery V20 fluorescence stereomicroscope fitted with a Tucsen FL20 microscope camera was used for histological imaging and either a Zeiss Axio Imager equipped with an Apotome 3 module or a Leica TCS SP-8 confocal microscope were used for imaging immunohistochemistry labelled sections.

### Edu labelling

To label proliferating cells, amputated fish were anesthetized in Tricaine and injected with 50 μl of 240 μg/mL of EdU solution daily. At 14dpa, fish were euthanized (excess of tricaine), the hearts were collected, and processed for immunohistochemistry. Following imaging, EdU + cardiomyocytes were counted using IMARIS software. *T*-test statistical analysis was performed using GraphPad Prism.

### RNA sequencing

Adult fish were anesthetized in Tricaine. Each group (*trpc6^−/−^* and *trpc6a^+/+^*) consists of 3 biological replicates of 5 pooled hearts. For each replicate 5 hearts were pooled and RNA was extracted using Trizol/choloform. Using DESeq2, a comparison of gene expression between the groups of samples was performed. The Wald test was used to generate *p*-values and log2 fold changes. Genes with an adjusted *p*-value <0.05 and absolute log2 fold change >1 were called as differentially expressed genes.

### Cardiomyocyte isolation and cyclic stretch

For each group (*trpc6a^+/+^* no stretch-3 groups, *trpc6a^+/+^* stretched-3 groups, *trpc6a^−/−^* no stretch-3 groups, *trpc6a*^−/−^ stretched-3 groups), 5 hearts were collected and pooled. Cardiomyocytes were isolated as previously described (29). Cardiomyocytes were plated on BioFlex® culture plates coated with poly lysine and centrifuged briefly (400 G/1 min). Following a period of 2 h to allow the cardiomyocytes to attach, a cyclic stretch protocol was applied for 24 h (Sine, 16% elongation, 0.5 Hz) with the Flexcell Tension System (FX-6000 T, Flexcell®) at 28°C and with 5% CO_2_.

### Real-time quantitative PCR

RNA was extracted from isolated cardiomyocytes using Trizol/chloroform. cDNA was obtained after reverse transcription using a First strand cDNA synthesis RT-PCR kit(Roche) and quantitative PCR was performed using SYBR Green (Roche) and a LightCycler 480 system (Roche). The primer sequences used are as follow:

*tubulin alpha* Forward: 5’ CGGCCAAGCAACACTACTAGA 3’

*tubulin alpha* Reverse: 5’ AGTTCCCAGCAGGCATTG 3’

*fosl1a* Forward: 5’ AAGGGAACGCAACAAAATGG 3’

*fosl1a* Reverse: 5’ AGCTTCTCCTTTTCCTTCTGG 3’

*nppb* Forward: 5’ TCCTCAGCGTTCAACACATG 3’

*nppb* Reverse: 5’ CCGCCTTTACTTCTCTTTCCG 3’

## Data Availability

The original contributions presented in the study are publicly available. This data can be found here: ArrayExpress (https://www.ebi.ac.uk/biostudies/arrayexpress#) accession E-MTAB-13603.

## Supplementary material

SUPPLEMENTARY FIGURE S1

Trpc6a regulates the expression of the AP1 transcription complex gene *fosl1a* during cardiac regeneration. (**A**) Table showing the difference in expression of *fosl1a* between *trpc6a^+/+^* sham hearts and *trpc6a^+/+^* 7dpa hearts and *trpc6a^−/−^* sham hearts and *trpc6a^−/−^* 7dpa hearts, note the significant increase in expression of *fosl1a* in the *trpc6a^+/+^* hearts. (**B**) *Adult mouse myocardial infarction.* Re-analysed UMAP plots depicting the expression *Fosl1* in uninjured sham conditions vs. 1, 7 and 14 days post myocardial infarction (MI). (**C**) Violin plots showing the expression of *Fosl1* at different time points after injury (sham, 1,7 and 14 days post myocardial infarction (d pMI) in cardiomyocytes (ns = not significant).

SUPPLEMENTARY FIGURE S2

Trpc6a regulates the expression of *nfatc2b* during cardiac regeneration. (**A**) Table showing the difference in expression of *nfatc2b* between *trpc6a^+/+^* sham hearts and *trpc6a^+/+^* 7dpa hearts and *trpc6a^−/−^* sham hearts and *trpc6a^−/−^* 7dpa hearts, note the significant increase in expression of *nfatc2b* in the *trpc6a^+/+^* hearts. (**B**) *Adult mouse myocardial infarction.* Re-analysed UMAP plots depicting the expression *Nfatc2* in uninjured sham conditions vs. 1, 7 and 14 days post myocardial infarction (MI). (**C**) Violin plots showing the expression of *Nfatc2* at different time points after injury (sham, 1,7 and 14 days post myocardial infarction (d pMI) in cardiomyocytes (ns = not significant).

SUPPLEMENTARY FIGURE S3

Trpc6a regulates the expression of the calcineurin/nfat target gene *nppb* during cardiac regeneration. (**A**) Table showing the difference in expression of *nppb* between *trpc6a^+/+^* sham hearts and *trpc6a^+/+^* 7dpa hearts and *trpc6a^−/−^* sham hearts and *trpc6a^−/−^* 7dpa hearts, note the significant increase in expression of *nppb* in the *trpc6a^+/+^* hearts. (**B**) *Adult mouse myocardial infarction.* Re-analysed UMAP plots depicting the expression *Nppb* in uninjured sham conditions vs. 1, 7 and 14 days post myocardial infarction (MI). (**C**) Violin plots showing the expression of *Nppb* at different time points after injury (sham, 1,7 and 14 days post myocardial infarction (d pMI) in cardiomyocytes. *P* values were adjusted using the Benjamini-Hochberg correction **** *P < 0.001*.

SUPPLEMENTARY FIGURE S4

The expression of *trpc6a* increases during cardiac regeneration. (**A**) Table showing the difference in expression of *trpc6a* between *trpc6a^+/+^* sham hearts and *trpc6a^+/+^* 7dpa hearts and *trpc6a^−/−^* sham hearts and *trpc6a^−/−^* 7dpa hearts, note the significant increase in expression of *trpc6a* in the *trpc6a^+/+^* hearts. (**B**) Immunohistochemistry using an anti-Trpc6 antibody (green) and DAPI (blue) on regenerating 7dpa *trpc6a^+/+^* hearts. White dashed line indicates the border zone between the myocardium and the injury site. (**C**) Higher magnification of the region denoted by the white square in (**B**) The yellow dashed circle highlights cardiomyocytes proximal to the border zone. The red dashed circle highlights cardiomyocytes distal to the border zone. (**D**) *Adult mouse myocardial infarction.* Re-analysed UMAP plots depicting the expression *Trpc6* in uninjured sham conditions vs. 1, 7 and 14 days post myocardial infarction (MI). (**C**) Violin plots showing the expression of *Trpc6* at different time points after injury (sham, 1,7 and 14 days post myocardial infarction (d pMI) in cardiomyocytes (ns = not significant).

SUPPLEMENTARY FIGURE S5

Trpc6a does not significantly regulate the expression of *nppb* in response to mechanical stretch. Relative expression of *nppb* in unstretched *trpc6a^+/+^* and *trpc6a^−/−^* cardiomyocytes. (**B**) Relative expression of *nppb* in *trpc6a^+/+^* and *trpc6a^−/−^* cardiomyocytes subjected to cyclic stretch.
