# Supplementary figures and images for: The ion channel Trpc6a regulates the cardiomyocyte regenerative response to mechanical stretch
Source: Front Cardiovasc Med. 2024 Jan 8;10:1186086. doi: 10.3389/fcvm.2023.1186086 (PMC10801195; doi:10.3389/fcvm.2023.1186086)

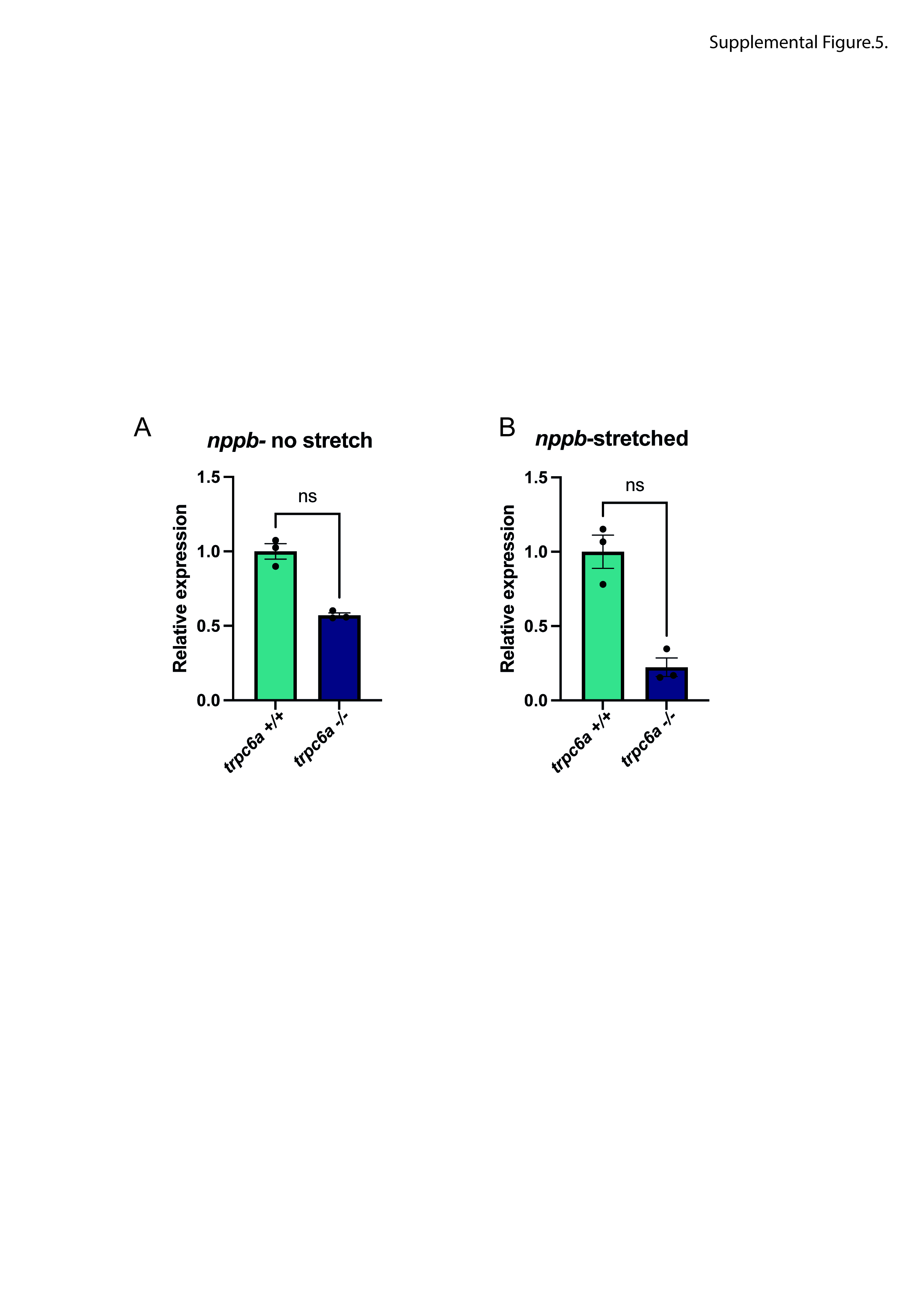

Supplement: Supplementary file 5 [file Image5.tif]
